# Supplementary material for: Characterizing spatiotemporal white matter hyperintensity pathophysiology in vivo to disentangle vascular and neurodegenerative contributions
Source: Nat Commun. 2026 Mar 31;17:4623. doi: 10.1038/s41467-026-70832-2 (PMC13199390; doi:10.1038/s41467-026-70832-2)
Supplement: Supplementary file 1 — Supplementary Information [file 41467_2026_70832_MOESM1_ESM.pdf]

Supplementary Material

Characterizing spatiotemporal white matter hyperintensity pathophysiology in vivo to disentangle vascular and neurodegenerative contributions

Olivier Parent, Zaki Alasmar, Sophia Osborne, Aurélie Bussy, Manuela Costantino, Jérémie P. Fouquet, Daniela Quesada, Alexandre Pastor-Bernier, Alfonso Fajardo-Valdez, Alexa Pichet-Binette, Ann McQuarrie, Josefina Maranzano, Gabriel A. Devenyi, Christopher J. Steele, Sylvia Villeneuve, the PREVENT-AD Research Group, the Alzheimer's Disease Neuroimaging Initiative (ADNI), Mahsa Dadar & M. Mallar Chakravarty

Supplementary Methods 3

Supplementary Methods 1. MRI acquisition parameters 3

Supplementary Methods 2. Processing of microstructural markers 4

Supplementary Tables 6

Supplementary Table 1. UK Biobank descriptive statistics. 6

Supplementary Table 2. ADNI descriptive statistics. 7

Supplementary Figures 8

Supplementary Figure 1. Step-by-step exclusions. 8

Supplementary Figure 2. Prevalence maps of WMH and NAWM 9

Supplementary Figure 3. Informing the choice of WMH and NAWM prevalence thresholds with voxel-wise standardized mean errors 10

Supplementary Figure 4. Choice of the number of spatial components and four-cluster solution. 11

Supplementary Figure 5. Variance explained by the clustering solution for each microstructural measure. 12

Supplementary Figure 6. Spatial clusters by severity of WMH burden. 13

Supplementary Figure 7. Spatial clusters by sex. 14

Supplementary Figure 8. Final WMH parcellation. 15

Supplementary Figure 9. Determining z-score thresholds for SuStaIn. 16

Supplementary Figure 10. Absence of sub-trajectories of pathophysiological cascades... 17

Supplementary Figure 11. Visualizing the uncertainty in SuStaIn temporal sequences.... 18

Supplementary Figure 12. Relationships between pathophysiological estimates and WMH volume. 19

Supplementary Figure 13. Descriptive distributions of diagnostic groupings. 20

Supplementary Figure 14. WMH pathophysiology in high genetic risk individuals using different polygenic risk score cut-off values. 21

Supplementary Figure 15. Interactions between sex and diseases on WMH pathophysiology. 22

Supplementary Figure 16. WMH pathophysiological effects in amyloid-positive individuals from ADNI. 23

Supplementary Figure 17. Differences in WMH pathophysiology across disorders: parcellation excluding rare WMH voxels. 24

Supplementary Figure 18. Performance of machine learning models classifying stroke and

cognitive impairment 25
Supplementary References 27

Supplementary Methods

Supplementary Methods 1. MRI acquisition parameters

MRI acquisition parameters for UK Biobank

1. **T1-weighted**: Sagittal 3D MPRAGE; in-plane acceleration factor (R) = 2; inversion time (TI) = 880 ms; repetition time (TR) = 2000 ms; resolution = 1 x 1 x 1 mm
2. **T2-weighted Fluid-attenuated inversion recovery (FLAIR)**: Sagittal 3D SPACE; R = 2; partial Fourier (PF) = 7/8; fat saturation; TI = 1800 ms; TR = 5000 ms; elliptical k-space scanning; resolution = 1.05 x 1 x 1 mm
3. **Diffusion-weighted imaging (DWI)**: SE-EPI; multiband factor (MB) = 3; R = 1; TE = 92 ms; TR = 3600 ms; PF = 6/8; fat saturation; b-values: 5 x b = 0 s/mm2, 50 x b = 1000 s/mm2, 50 x b = 2000 s/mm2 (100 distinct diffusion directions); phase-encoding reversed data acquired; resolution = 2 x 2 x 2 mm
4. **Susceptibility-weighted imaging (SWI)**: Axial 3D GRE; R = 2, PF = 7/8; TE1 = 9.4 ms; TE2 = 20 ms; TR = 27 ms; resolution = 0.8 x 0.8 x 3 mm

MRI acquisition parameters for ADNI

1. **T1-weighted**: MPRAGE; acceleration factor = 2; TE = min full echo; TR = 2300 ms; TI = 900 ms; resolution = 1 x 1 x 1 mm
2. **T2-weighted FLAIR:** 3D FLAIR; TE = 119 ms; TR = 4800 ms; TI = 1650 ms; resolution = 1.2 x 1 x 1 mm
3. **DWI**: TE = 71 ms; TR = 3300 ms; b = 500, 1000, 2000 s/mm2 (112 distinct diffusion directions); resolution = 2 x 2 x 2 mm

Supplementary Methods 2. Processing of microstructural markers

Microstructural markers were processed by the UK Biobank team.[1,2] The multi-shell diffusion-weighted images were corrected for susceptibility artifacts using the *topup* FSL command,[3] eddy currents, head motion, and outliers using the *eddy* command,[4] and gradient distortions.[5] The cleaned first diffusion shell was used as input to the *DTIFIT* tool to generate diffusion tensor imaging (DTI) markers.[6] The multi-shell acquisition allowed for more advanced modeling techniques of the diffusion signal. Neurite orientation dispersion and density imaging (NODDI) markers were generated with the *AMICO* tool.[7,8]

The susceptibility-weighted images were saved as magnitude and phase images separately for each coil. Magnitude data was combined across coils using a sum-of-squares calculation, and from this data, T2* is calculated as the inverse of the log ratio of the two echo times scaled by the echo time difference.[1] Phase data was combined across coils using *MCPC-3D-S*, which removes phase cancellation artifacts from each echo.[9] This data underwent phase unwrapping using a Laplacian algorithm,[10,11] background field removal using *V-SHARP*,[12] and brain mask erosion to exclude voxels with low phase reliability. Dipole inversion using *iLSQR* was then used to estimate quantitative susceptibility mapping (QSM) maps,[13] which were further normalized by the subject-wise median value in the ventricles. The complete detailed processing for the DTI, NODDI, and T2* markers is available in Alfaro-Almagro et al., 2018,[1] and for QSM in Wang et al., 2022.[2]

In ADNI, we performed image processing for microstructural maps ourselves, matching the processing steps of UKB whenever possible. Since there was no phase-encoding reversed data acquired for the ADNI DWI acquisition, we corrected diffusion distortion artifacts using the Synthesized b0 for diffusion distortion correction (*Synb0-DisCo*) tool, which used deep learning to generate an undistorted b0 image using information from the T1w image.[14] This

data was then fed into *topup*, *eddy*, *DTIFIT*, and *AMICO* while matching input parameters from the UKB processing.

Supplementary Tables

		Complete sample (*n*=39,676)	After exclusions (*n*=32,526)
**Age**			
	Mean (s.d.; min-max)	63.62 (7.55; 44 - 82)	63.52 (7.49; 45 - 81)
**Sex**			
	Female/Male	20,990 (52.9%)/ 18,676 (47.1%)	17,382 (53.4%)/ 15,144 (46.6%)
**Ethnicity**			
	White	38,392 (96.8%)	31,533 (96.9%)
	Black	254 (0.6%)	197 (0.6%)
	Asian	535 (1.3%)	408 (1.3%)
	Mixed	178 (0.4%)	147 (0.5%)
	Other	201 (0.5%)	155 (0.5%)
**Education**			
	7 years	2,481 (6.3%)	1,979 (6.1%)
	10 years	5,144 (13%)	4,229 (13%)
	13 years	2,383 (6%)	1,938 (6%)
	15 years	4,411 (11.1%)	3,645 (11.2%)
	19 years	5,804 (14.6%)	4,757 (14.6%)
	20 years	19,304 (48.7%)	15,868 (48.8%)

Supplementary Table 1. UK Biobank descriptive statistics.

Demographic statistics in the UK Biobank before and after exclusions.

		Complete sample (*n*=212)	Cognitively normal (*n*=125)	Mild cognitive impairment (*n*=66)	Alzheimer's disease (*n*=21)
**Age**					
	Mean (s.d.; min-max)	73.41 (8.44; 51 - 93)	72.49 (8.64; 51 - 92)	73.86 (7.99; 55 - 93)	77.42 (7.62; 60 - 89)
**Sex**					
	Female/Male	123 (58%)/89 (42%)	83 (66.4%)/42 (33.6%)	30 (45.5%)/36 (54.5%)	10 (47.6%)/11 (52.4%)
**Ethnicity***					
	White	167 (78.8%)	96 (76.8%)	55 (83.3%)	16 (76.2%)
	Black	29 (13.7%)	18 (14.4%)	7 (10.6%)	4 (19%)
	Asian	8 (3.8%)	6 (4.8%)	2 (3%)	0 (0%)
	Mixed	3 (1.4%)	1 (0.8%)	1 (1.5%)	1 (4.8%)
**Education years**					
	Mean (s.d.; min-max)	16.13 (2.43; 11 - 20)	16.52 (2.35; 11 - 20)	15.74 (2.45; 12 - 20)	15 (2.35; 12 - 18)
**Amyloid status***					
	Positive	70 (42.2%)	28 (28%)	26 (53.1%)	16 (94.1%)

Supplementary Table 2. ADNI descriptive statistics.

Demographic statistics in ADNI in the complete sample and by cognitive group. *Removing missing values

Supplementary Figures

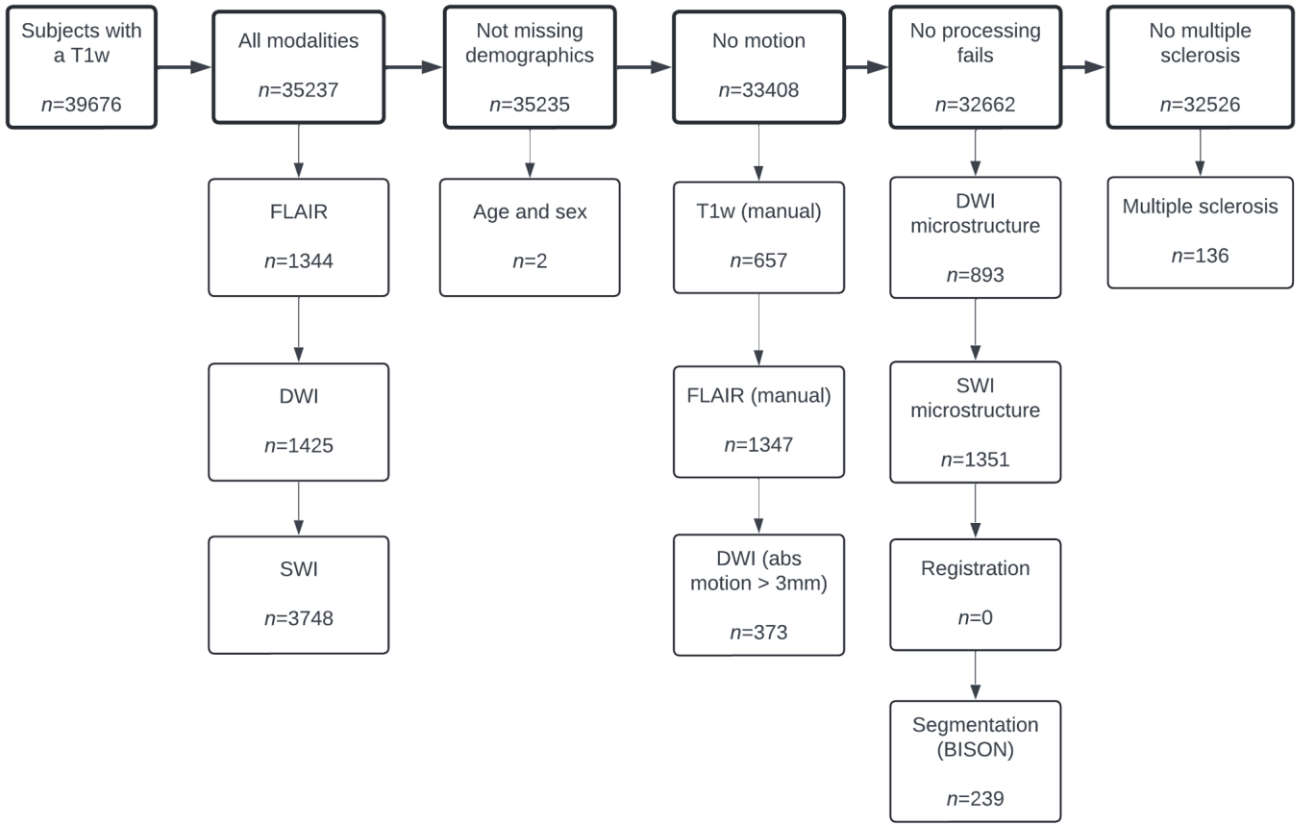

Supplementary Figure 1. Step-by-step exclusions.

Top rows: number of participants left after each exclusion step. Borrow rows: number of participants excluded for each criterion. Abbreviations: diffusion-weighted imaging (DWI), susceptibility-weighted imaging (SWI), T1-weighted (T1w), fluid-attenuated inversion recovery (FLAIR), BraIn SegmentatiON algorithm (BISON)

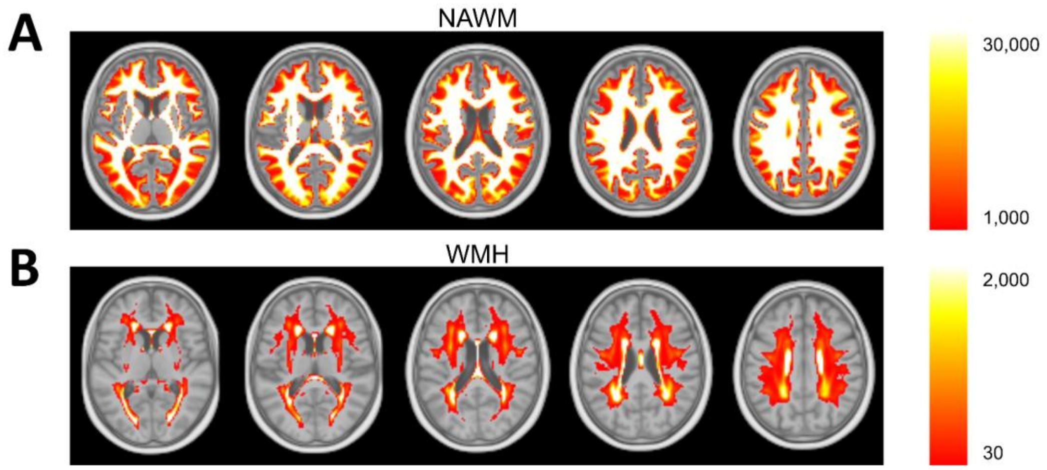

Supplementary Figure 2. Prevalence maps of WMH and NAWM

Prevalence across individuals of **A)** NAWM and **B)** WMH labelled voxels. WMH: white matter hyperintensities; NAWM: normal-appearing white matter.

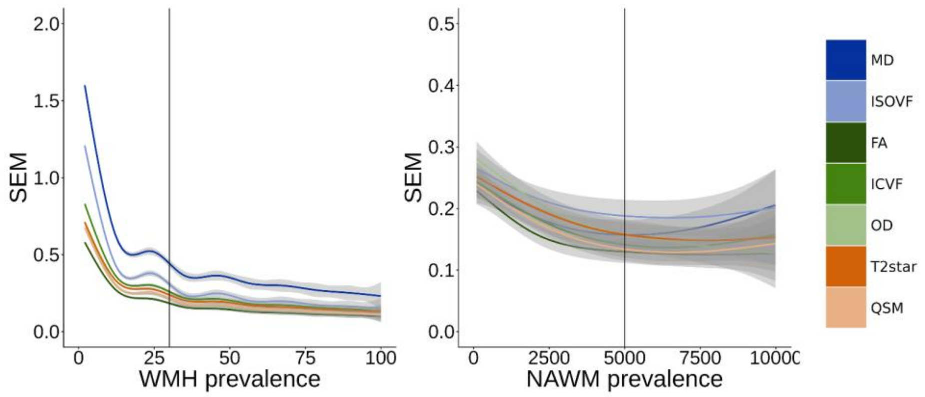

Supplementary Figure 3. Informing the choice of WMH and NAWM prevalence thresholds with voxel-wise standardized mean errors

For each marker, the standardized mean error (SEM) was plotted as a function of WMH or NAWM prevalence by fitting a generalized additive model using the *geom_smooth* function in R. Chosen thresholds are indicated with vertical black lines (WMH: 30; NAWM: 5000)

A

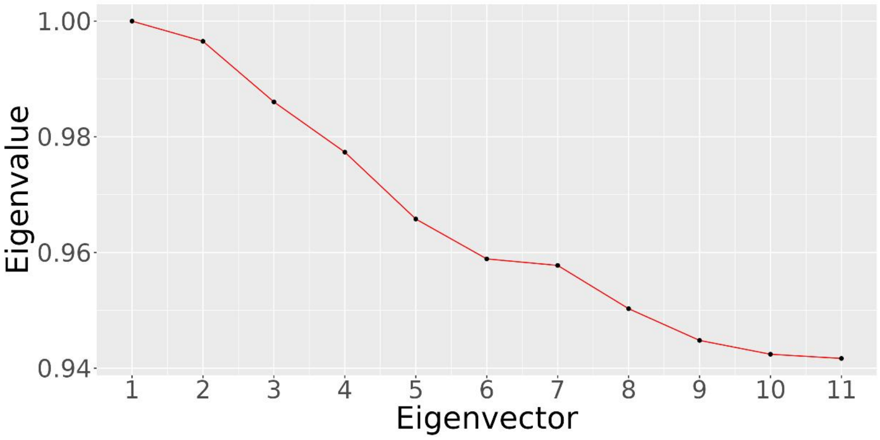

B

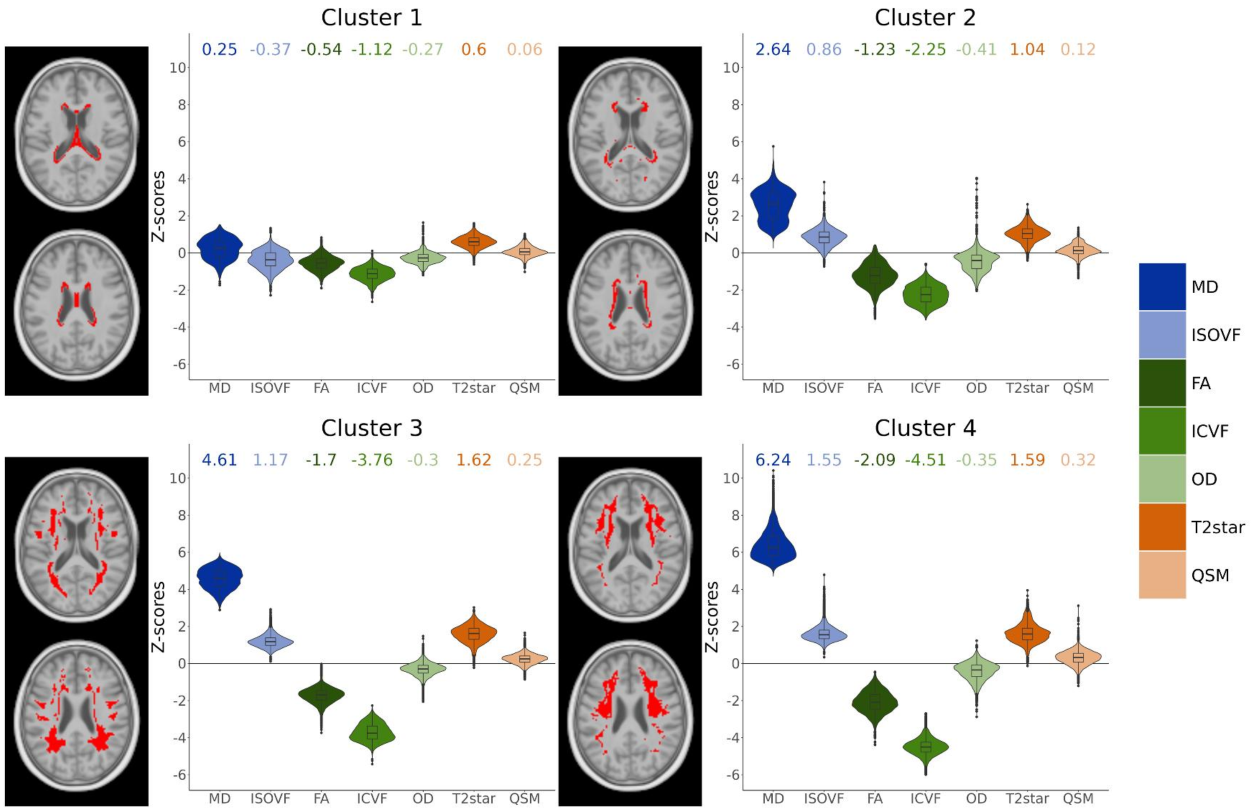

Supplementary Figure 4. Choice of the number of spatial components and four-cluster solution.

A) Eigengap graph to guide the choice of the number of clusters. According to this heuristic, the optimal number of clusters is 4. **B)** Spatial clusters for the four-cluster solution. Left: Voxels included in the cluster are indicated in red. Right: Pathophysiological distributions of the voxels included in the cluster. Median values of those distributions are shown at the top. These clusters are less spatially contiguous than the three-cluster solution, as clusters 1 and 2 are very similar. Source data are provided as a Source Data file. MD: mean diffusivity; FA: fractional anisotropy; ISOVF: isotropic volume fraction; ICVF: intra-cellular volume fraction; OD: orientation dispersion; QSM: quantitative susceptibility mapping.

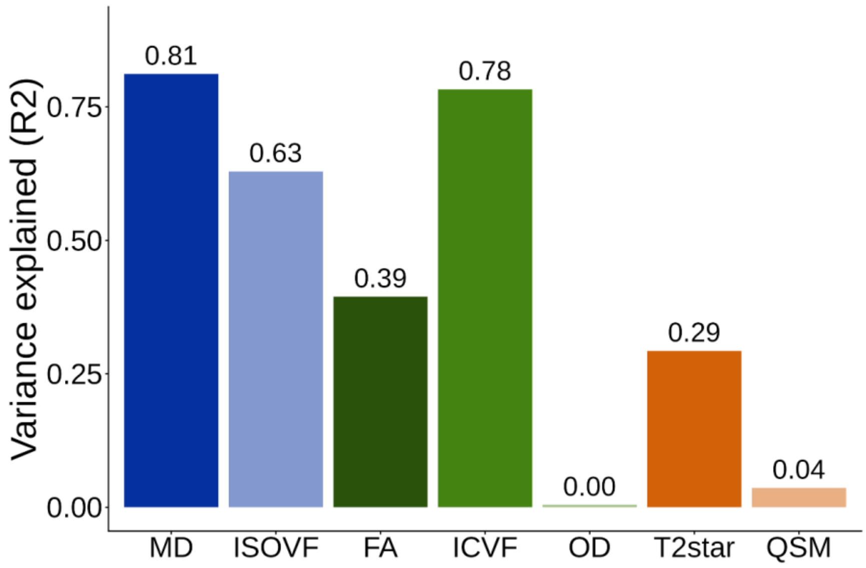

Supplementary Figure 5. Variance explained by the clustering solution for each microstructural measure.

For each microstructural measure, an ANOVA was used to calculate the variance explained (R^2) in the maps of between-subject averaged WMH pathophysiology (Figure 2A) by the spatial clustering solution (Figure 2B). Source data are provided as a Source Data file.

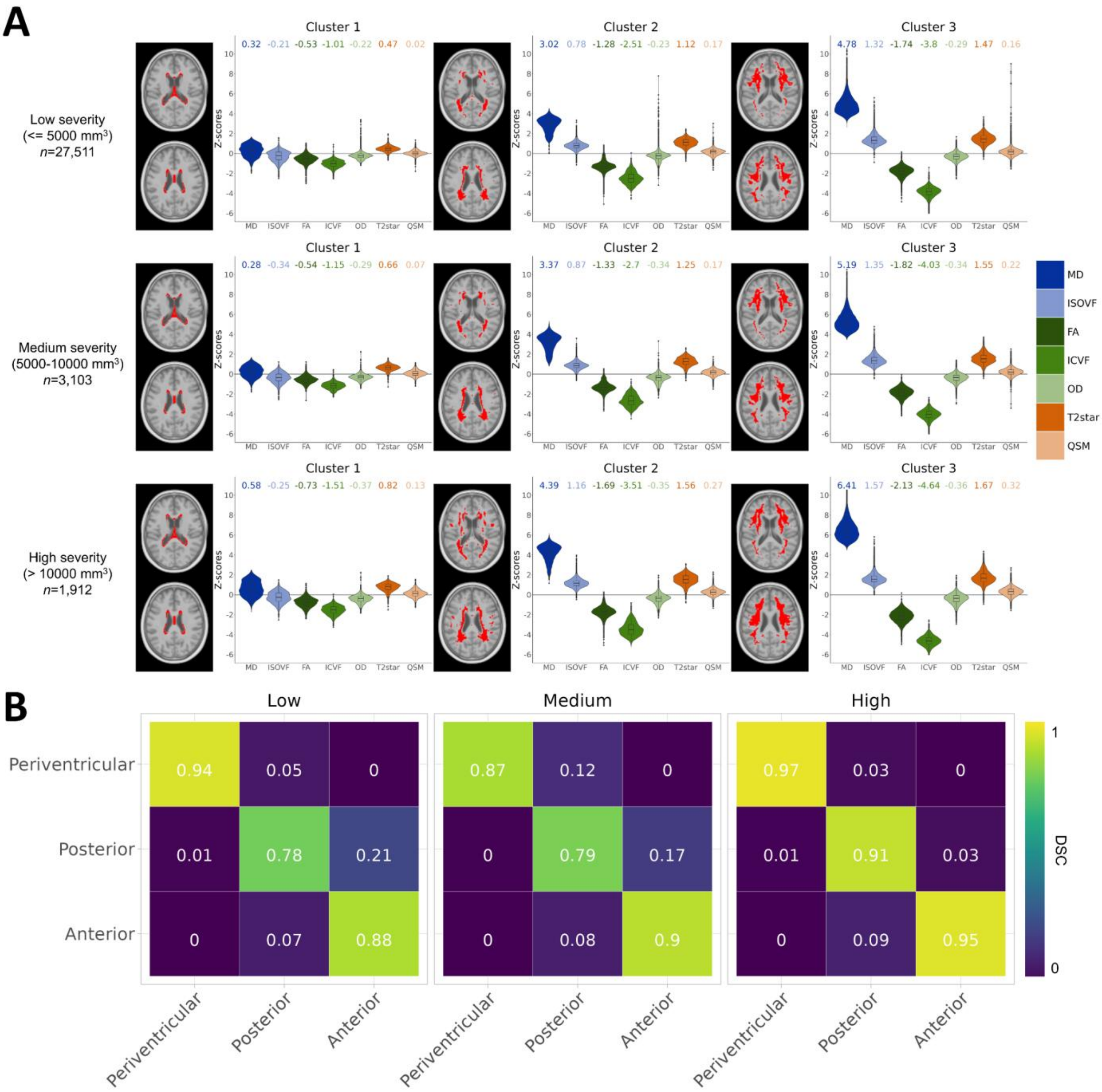

Supplementary Figure 6. Spatial clusters by severity of WMH burden.

A) Spatial clusters were calculated in different brackets of total WMH volumes. The sample size in each bracket is indicated. Only voxels with WMH prevalence >10 are included per group. Left: voxels included in the cluster are indicated in red. Right: Pathophysiological distributions of the voxels included in the cluster. Median values of those distributions are shown at the top. **B)** Dice similarity coefficients (DSCs) between the clusters in each severity bracket (x-axis) and the original clusters derived in the whole sample (y-axis). Only voxels included in the original and new clustering solutions are included in the calculation. Source data are provided as a Source Data file.

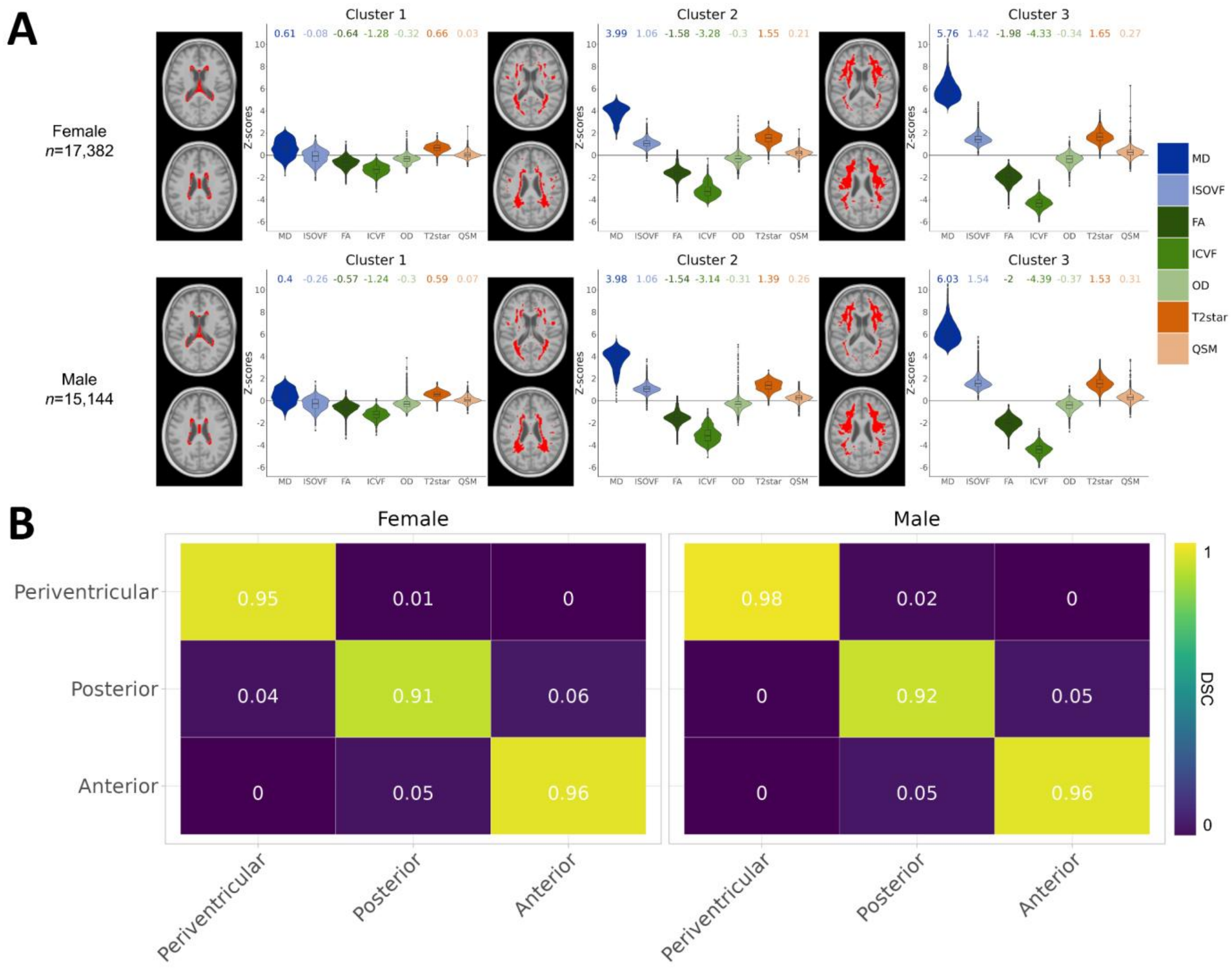

Supplementary Figure 7. Spatial clusters by sex.

A) Spatial clusters were calculated in females and males separately. The sample size for each sex is indicated. Only voxels with WMH prevalence >15 are included per group. Left: voxels included in the cluster are indicated in red. Right: Pathophysiological distributions of the voxels included in the cluster. Median values of those distributions are shown at the top. **B)** DSCs between the clusters calculated in females and males (x-axis) and the original clusters derived in the whole sample (y-axis). Only voxels included in the original and new clustering solutions are included in the calculation. Source data are provided as a Source Data file.

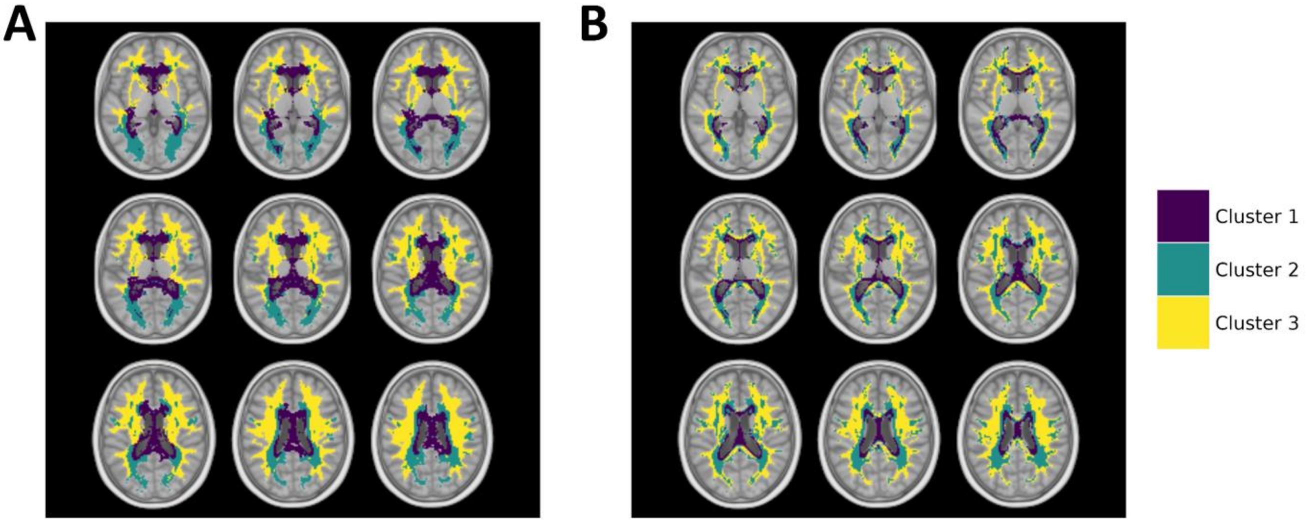

Supplementary Figure 8. Final WMH parcellation.

A) Three-cluster solution only including voxels with a high prevalence of WMHs (>30) and NAWM (>5000), which was then filled to include previously excluded voxels using a search area strategy. **B)** Direct clustering of all WMH voxels (WMH prevalence > 1). However, a NAWM prevalence threshold (>1000) was still necessary to ensure the stability of normative modeling.

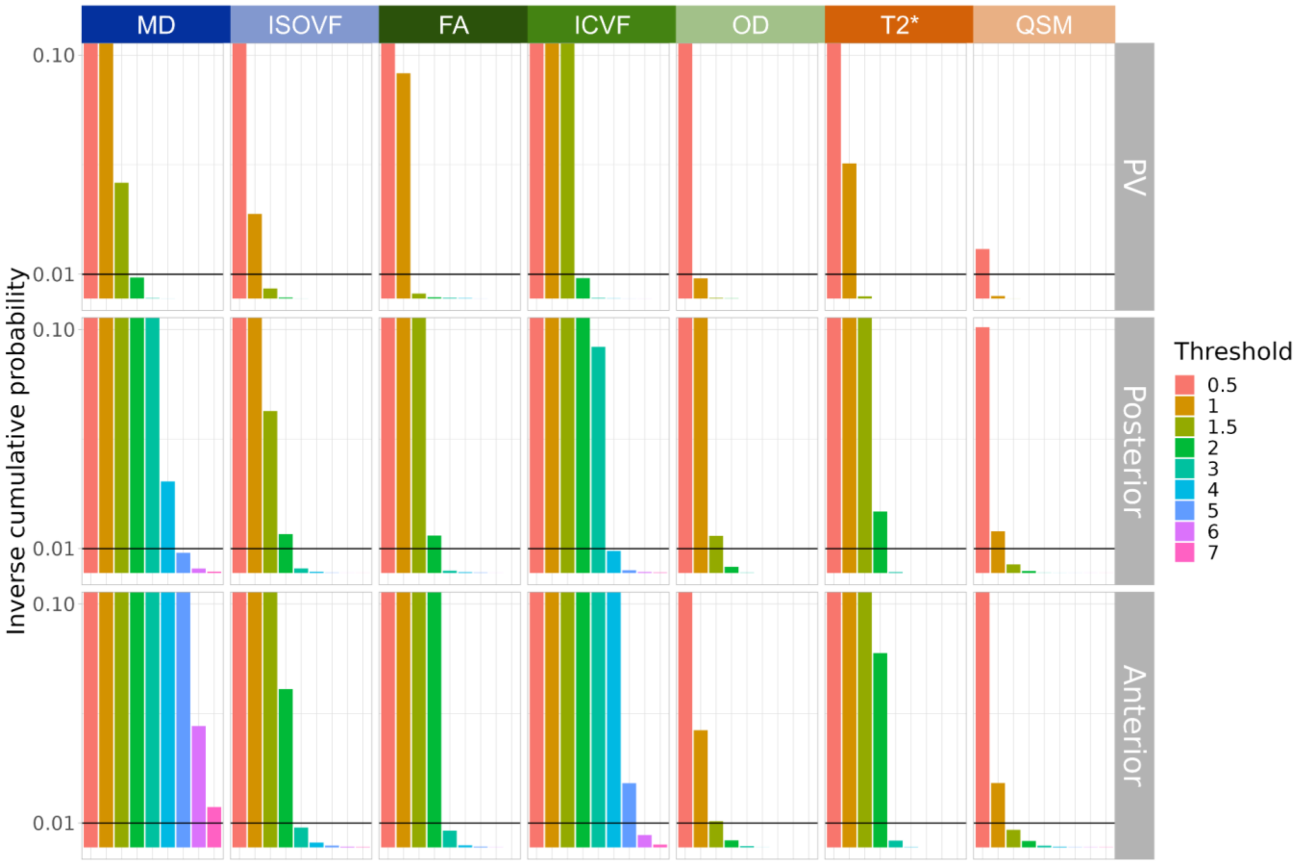

Supplementary Figure 9. Determining z-score thresholds for SuStaIn.

The inverse cumulative probabilities for every microstructural marker in every WMH region are shown. The maximum Subtype and Stage Inference (SuStaIn) input events are determined as the last thresholds reached by at least 1% of participants (last column above the black line). Source data are provided as a Source Data file. PV: Periventricular

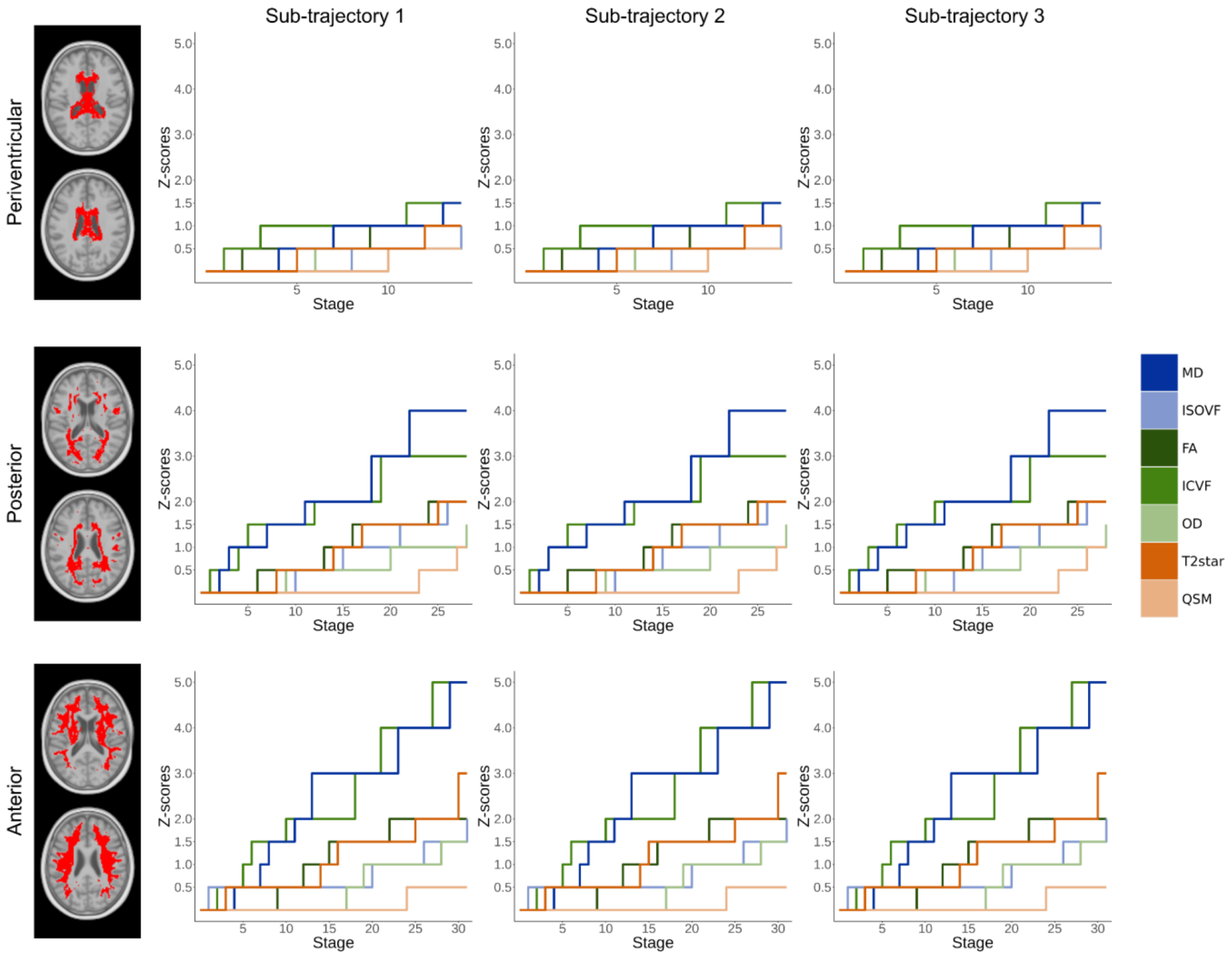

Supplementary Figure 10. Absence of sub-trajectories of pathophysiological cascades.

Within each spatial region (rows), SuStaIn was set to model three sub-trajectories (columns). Shown here are the winner-take-all trajectories. The x-axes represent a data-driven temporal axis of pathophysiological events (stages) and the y-axes represent the abnormality z-score thresholds. These sub-trajectories do not show meaningful differences when compared within regions. Source data are provided as a Source Data file.

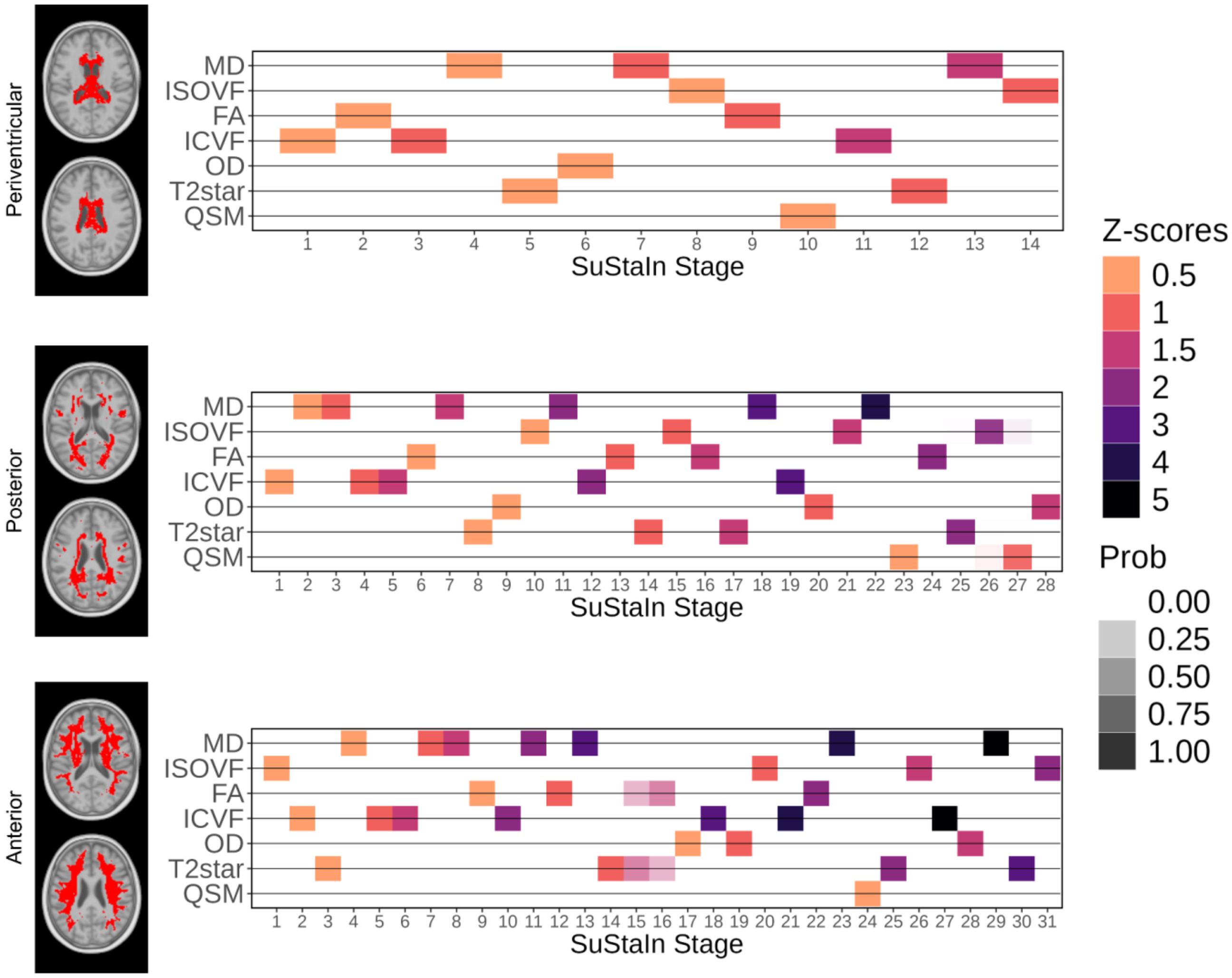

Supplementary Figure 11. Visualizing the uncertainty in SuStaIn temporal sequences.

Positional variance diagrams for SuStaIn trajectories. The opacity indicates the percentage of times that the pathophysiological event was placed at that stage across 10-fold cross-validation and 10,000 Monte Carlo Markov Chain resamples at each fold. Source data are provided as a Source Data file. Prob: probability.

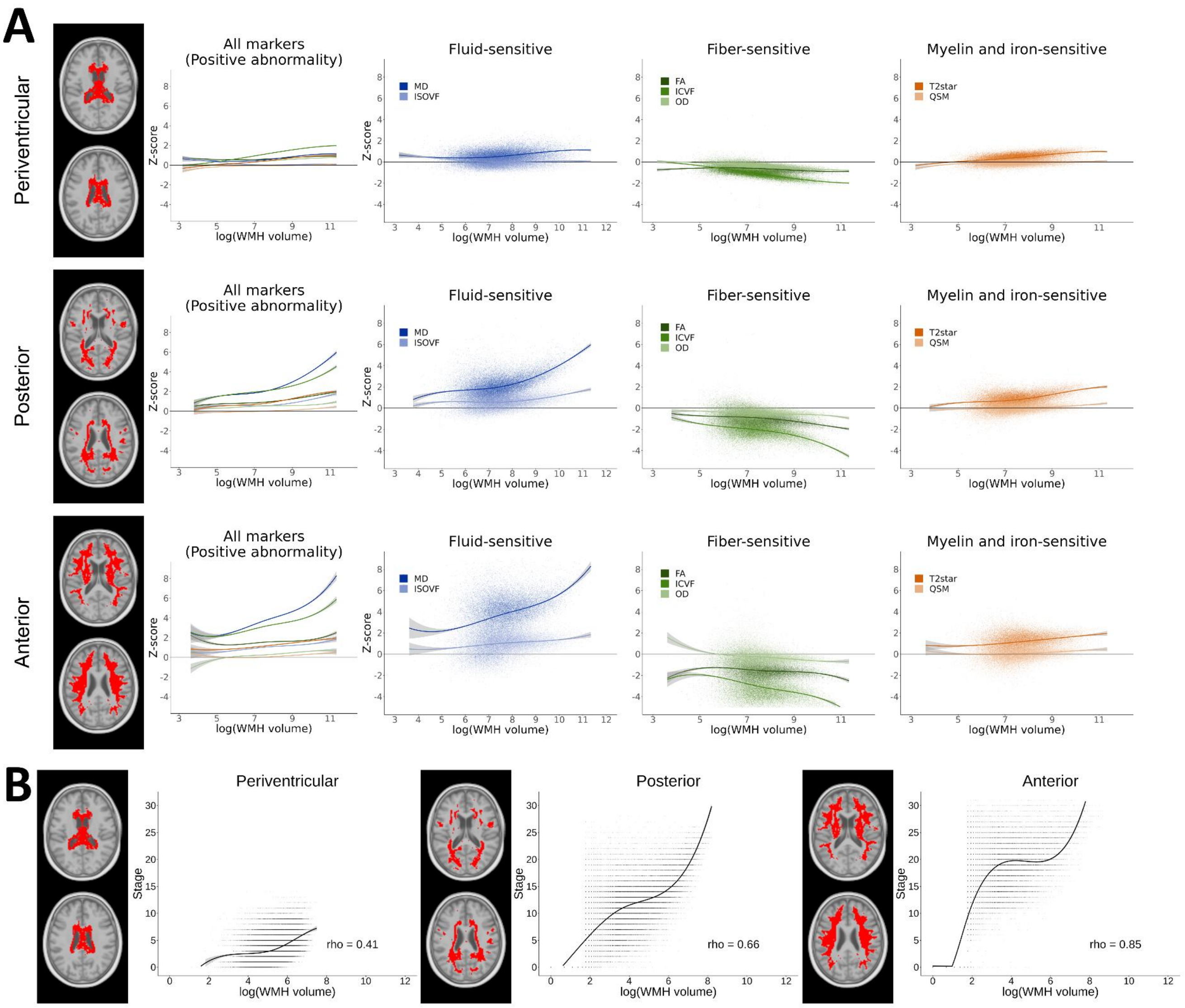

Supplementary Figure 12. Relationships between pathophysiological estimates and WMH volume.

A) The first graphs on the left show the trajectories of pathophysiological estimates relative to WMH volume (log-transformed) fit with fourth-order B-splines, with a higher positive abnormality indicative of worst WMH pathophysiology to be comparable with the SuStaIn results. The three graphs on the right show the trajectories by biological sensitivity, together with the distributions of the underlying data and keeping the original directionality of the measures. **B)** Associations between SuStaIn stages and log-transformed WMH volumes in each spatial cluster. Spearman correlation values are indicated (all $p<0.001$)

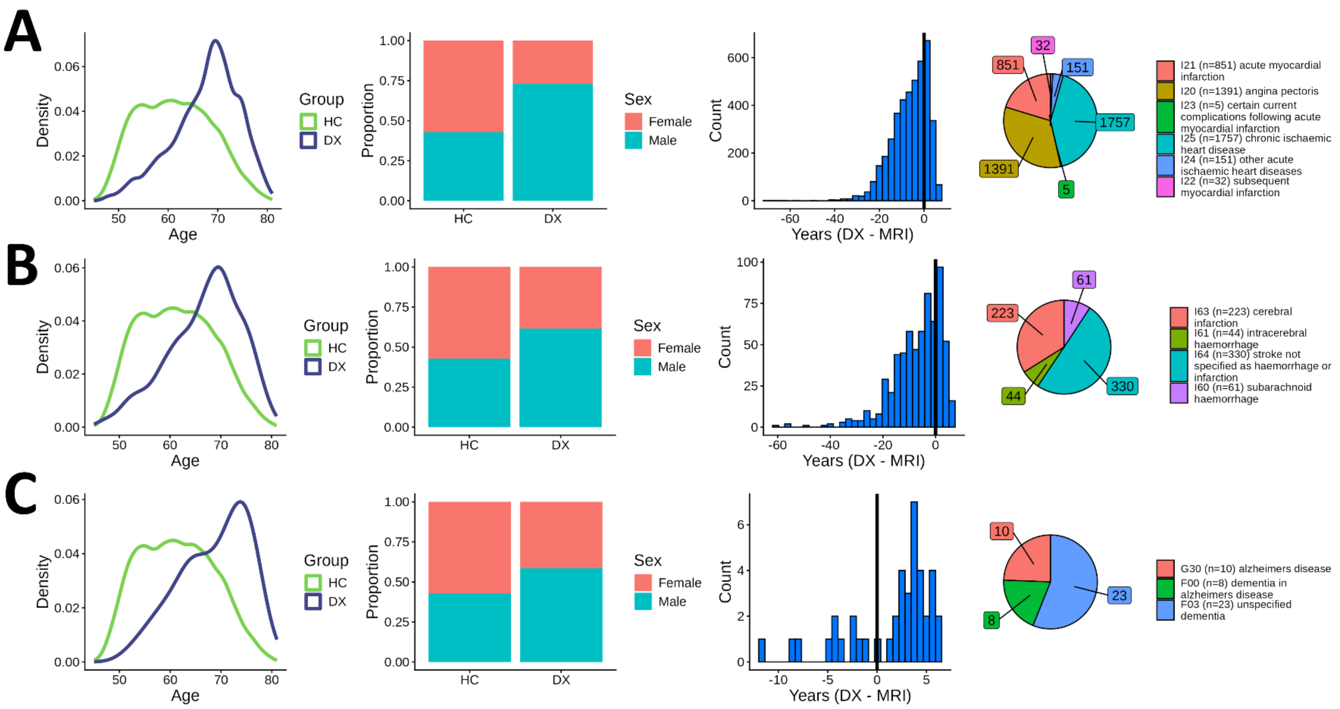

Supplementary Figure 13. Descriptive distributions of diagnostic groupings. Distributions of age and sex between cases and controls, timing of the diagnosis relative to the MRI visit date, and individual ICD-10 diagnoses. **A)** Ischemic heart diseases (*n*=2,414). **B)** Stroke (*n*=645). **C)** Dementia (excluding vascular dementia; *n*=47). HC: healthy controls; DX: diagnosis.

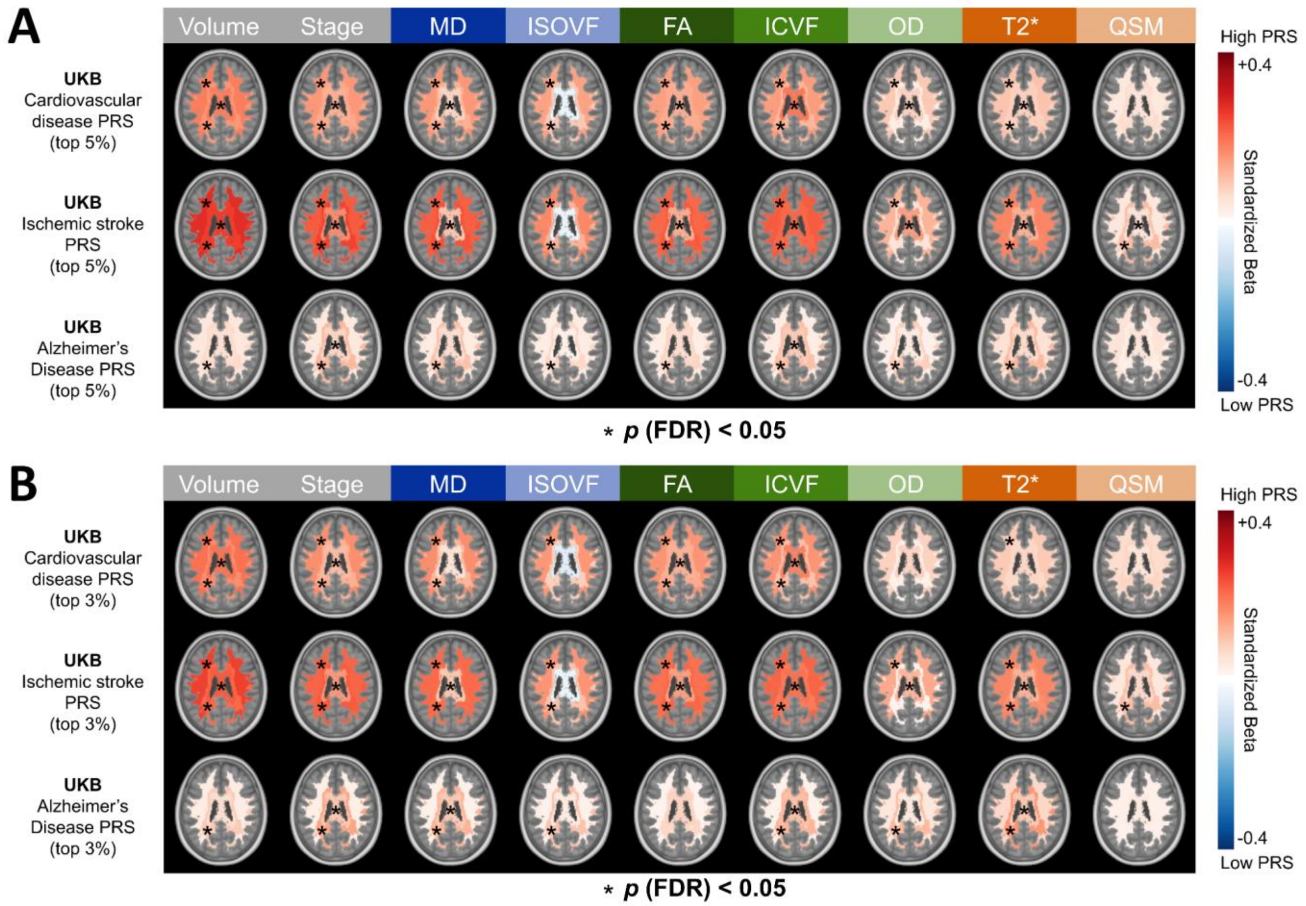

Supplementary Figure 14. WMH pathophysiology in high genetic risk individuals using different polygenic risk score cut-off values.

Effect size patterns of spatiotemporal WMHs pathophysiology comparing cases and controls according to genetic risk. The "case" group was determined using polygenic risk score (PRS) cut-off values of top 5% **(A)** and top 3% **(B)** to supplement the analysis in the main text, which used the value of top 1% (Figure 4B). Source data are provided as a Source Data file. UKB: UK Biobank; FDR: false discovery rate.

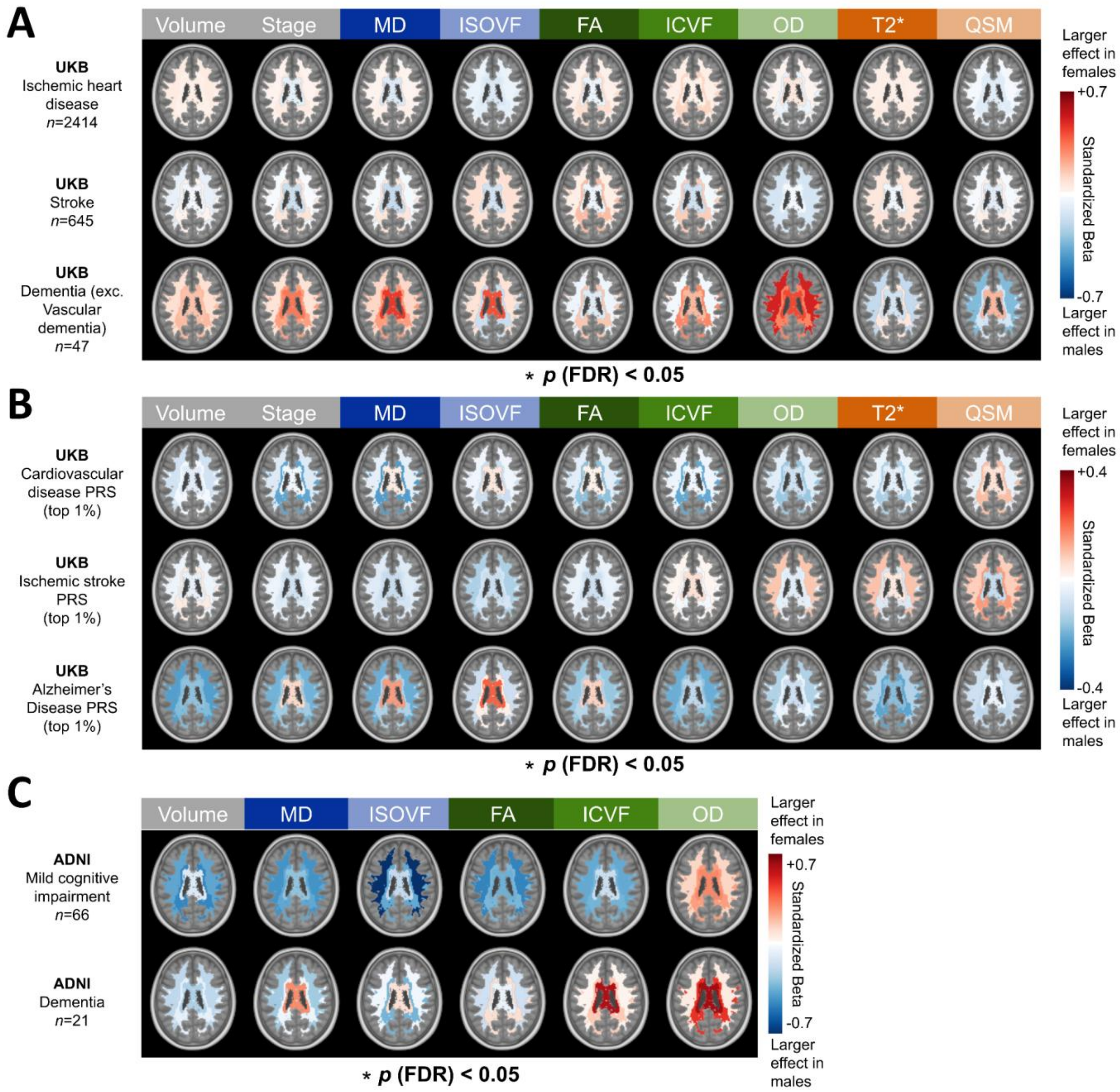

Supplementary Figure 15. Interactions between sex and diseases on WMH pathophysiology.

Effect size patterns of spatiotemporal WMHs pathophysiology comparing the effect of case/control differences across males and females in **A)** UK Biobank participants according to ICD-10 code groupings of ischemic heart disease, stroke, and dementia, **B)** UK Biobank participants at high genetic risk of cardiovascular disease, ischemic stroke, and Alzheimer's disease, and **C)** ADNI participants with mild cognitive impairment due to Alzheimer's disease and Alzheimer's disease dementia. Red colors indicate higher WMH burden in cases and blue colors indicate higher WMH burden in controls. Significant effects at the FDR-corrected $p<0.05$ level are indicated with black asterisks (no effects were significant). Source data are provided as a Source Data file. ADNI: Alzheimer's disease neuroimaging initiative.

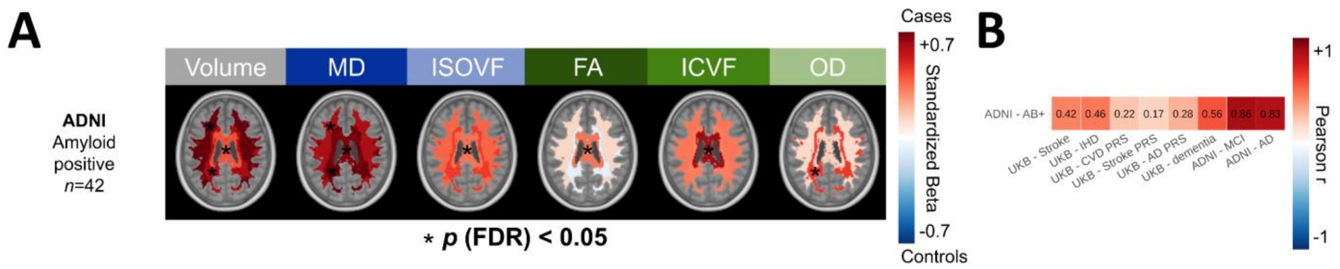

Supplementary Figure 16. WMH pathophysiological effects in amyloid-positive individuals from ADNI.

A) Effect sizes across WMH measures comparing amyloid-positive AD and MCI cases (*n*=42) to amyloid-negative cognitively healthy controls (*n*=70) in the ADNI dataset. Red colors indicate higher WMH burden in cases and blue colors indicate higher WMH burden in controls. Significant effects at the FDR-corrected $p<0.05$ level are indicated with black asterisks. **B)** Correlations between the observed effect size pattern and other computed patterns from the main analysis (Figure 4). Source data are provided as a Source Data file.

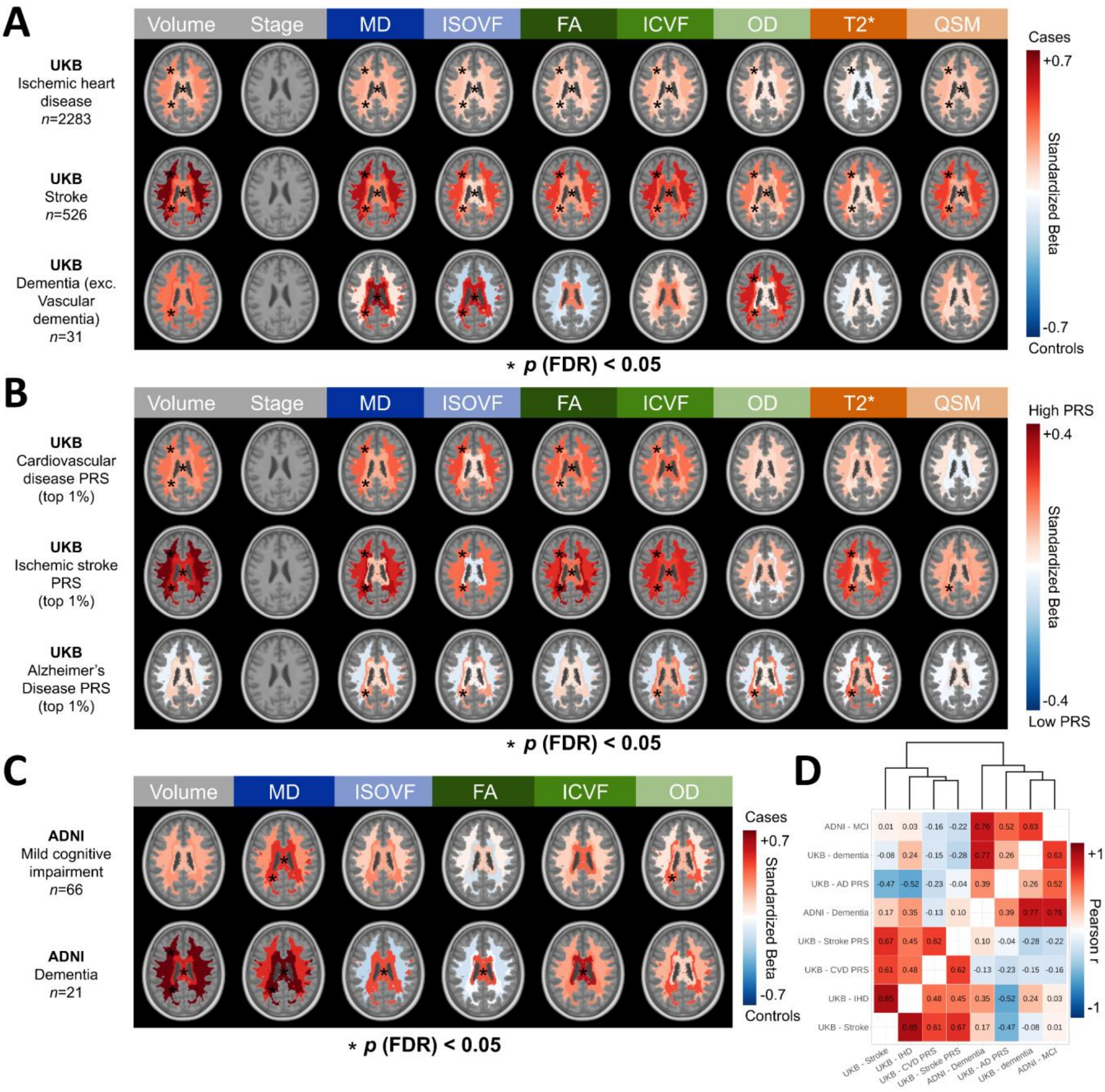

Supplementary Figure 17. Differences in WMH pathophysiology across disorders: parcellation excluding rare WMH voxels.

Results from Figure 4 were reproduced with a parcellation that did not include previously-excluded rare WMH voxels with our search area strategy. Effect size patterns of spatiotemporal WMHs pathophysiology comparing cases and controls in **A)** UK Biobank participants according to ICD-10 code groupings of ischemic heart disease, stroke, and dementia, **B)** UK Biobank participants at high genetic risk of cardiovascular disease, ischemic stroke, and Alzheimer's disease, and **C)** ADNI participants with mild cognitive impairment and dementia. Red colors indicate higher WMH burden in cases and blue colors indicate higher WMH burden in controls. Significant effects at the FDR-corrected $p<0.05$ level are indicated with black asterisks. **D)** Effect size patterns were correlated and clustered with hierarchical clustering. Source data are provided as a Source Data file.

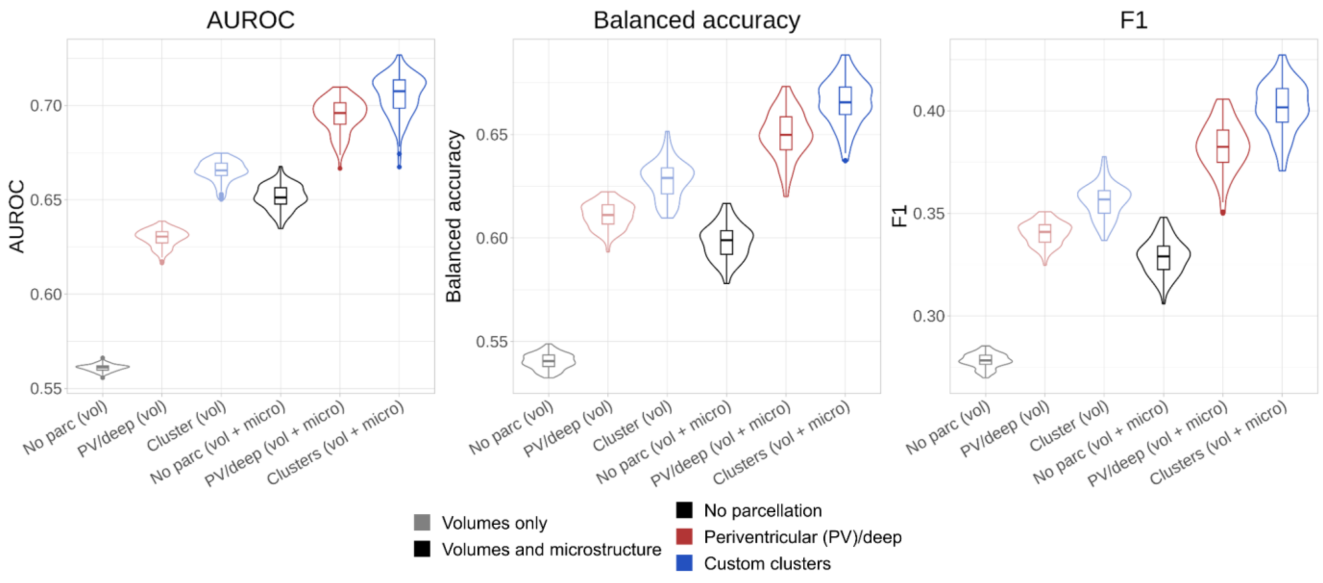

Supplementary Figure 18. Performance of machine learning models classifying stroke and cognitive impairment.

Lasso logistic regression models with different sets of predictors (x-axis) were used to classify individuals with stroke and cognitive impairment. Individuals with stroke diagnoses (n=642) from individuals with dementia or MCI diagnoses (n=127) were classified, combining data from UKB and ADNI after removing site effects with ComBat. Distributions of performance metrics are calculated using out-of-fold predictions with 5-fold cross-validation repeated 100 times. Medians, first and third quartiles are indicated with box plots. Source data are provided as a Source Data file. Parc: parcellation; vol: volume; PV: periventricular; micro: microstructure.

Supplementary References

1. Alfaro-Almagro, F. *et al.* Image processing and Quality Control for the first 10,000 brain imaging datasets from UK Biobank. *NeuroImage* **166**, 400–424 (2018).
2. Wang, C. *et al.* Phenotypic and genetic associations of quantitative magnetic susceptibility in UK Biobank brain imaging. *Nat. Neurosci.* **25**, 818–831 (2022).
3. Andersson, J. L. R., Skare, S. & Ashburner, J. How to correct susceptibility distortions in spin-echo echo-planar images: application to diffusion tensor imaging. *NeuroImage* **20**, 870–888 (2003).
4. Andersson, J. L. R. & Sotiropoulos, S. N. An integrated approach to correction for off-resonance effects and subject movement in diffusion MR imaging. *NeuroImage* **125**, 1063–1078 (2016).
5. Glasser, M. F. *et al.* The minimal preprocessing pipelines for the Human Connectome Project. *NeuroImage* **80**, 105–124 (2013).
6. Basser, P. J., Mattiello, J. & Lebihan, D. Estimation of the Effective Self-Diffusion *Tensor* from the NMR Spin Echo. *J. Magn. Reson. B* **103**, 247–254 (1994).
7. Zhang, H., Schneider, T., Wheeler-Kingshott, C. A. & Alexander, D. C. NODDI: Practical in vivo neurite orientation dispersion and density imaging of the human brain. *NeuroImage* **61**, 1000–1016 (2012).
8. Daducci, A. *et al.* Accelerated Microstructure Imaging via Convex Optimization (AMICO) from diffusion MRI data. *NeuroImage* **105**, 32–44 (2015).
9. Eckstein, K. *et al.* Computationally Efficient Combination of Multi-channel Phase Data From Multi-echo Acquisitions (ASPIRE). *Magn. Reson. Med.* **79**, 2996–3006 (2018).
10. Wu, B., Li, W., Avram, A. V., Gho, S.-M. & Liu, C. Fast and tissue-optimized mapping of magnetic susceptibility and T2* with multi-echo and multi-shot spirals. *NeuroImage* **59**, 297–305 (2012).

11. Schofield, M. A. & Zhu, Y. Fast phase unwrapping algorithm for interferometric applications. *Opt. Lett.* **28**, 1194–1196 (2003).

12. Schweser, F., Deistung, A., Lehr, B. W. & Reichenbach, J. R. Quantitative imaging of intrinsic magnetic tissue properties using MRI signal phase: An approach to in vivo brain iron metabolism? *NeuroImage* **54**, 2789–2807 (2011).

13. Li, W. *et al.* A method for estimating and removing streaking artifacts in quantitative susceptibility mapping. *NeuroImage* **108**, 111–122 (2015).

14. Schilling, K. G. *et al.* Synthesized b0 for diffusion distortion correction (Synb0-DisCo). *Magn. Reson. Imaging* **64**, 62–70 (2019).
