# Supplementary material for: Characterizing spatiotemporal white matter hyperintensity pathophysiology in vivo to disentangle vascular and neurodegenerative contributions
Source: Nat Commun. 2026 Mar 31;17:4623. doi: 10.1038/s41467-026-70832-2 (PMC13199390; doi:10.1038/s41467-026-70832-2)
Supplement: Supplementary file 2 — Reporting Summary [file 41467_2026_70832_MOESM2_ESM.pdf]

nature portfolio

Corresponding author(s): Olivier Parent, Mahsa Dadar, M. Mallar Chakravarty

Last updated by author(s): Oct 20, 2025

Reporting Summary

Nature Portfolio wishes to improve the reproducibility of the work that we publish. This form provides structure for consistency and transparency in reporting. For further information on Nature Portfolio policies, see our Editorial Policies and the Editorial Policy Checklist.

Statistics

For all statistical analyses, confirm that the following items are present in the figure legend, table legend, main text, or Methods section.

n/a	Confirmed	
☐	☒	The exact sample size (*n*) for each experimental group/condition, given as a discrete number and unit of measurement
☐	☒	A statement on whether measurements were taken from distinct samples or whether the same sample was measured repeatedly
☐	☒	The statistical test(s) used AND whether they are one- or two-sided<br>*Only common tests should be described solely by name; describe more complex techniques in the Methods section.*
☐	☒	A description of all covariates tested
☐	☒	A description of any assumptions or corrections, such as tests of normality and adjustment for multiple comparisons
☐	☒	A full description of the statistical parameters including central tendency (e.g. means) or other basic estimates (e.g. regression coefficient) AND variation (e.g. standard deviation) or associated estimates of uncertainty (e.g. confidence intervals)
☐	☒	For null hypothesis testing, the test statistic (e.g. *F*, *t*, *r*) with confidence intervals, effect sizes, degrees of freedom and *P* value noted<br>*Give P values as exact values whenever suitable.*
☒	☐	For Bayesian analysis, information on the choice of priors and Markov chain Monte Carlo settings
☒	☐	For hierarchical and complex designs, identification of the appropriate level for tests and full reporting of outcomes
☐	☒	Estimates of effect sizes (e.g. Cohen's *d*, Pearson's *r*), indicating how they were calculated

Our web collection on statistics for biologists contains articles on many of the points above.

Software and code

Policy information about availability of computer code

Data collection	No software was used for collection of data in this study.
Data analysis	MRI data was processed using minc-toolkit/v1.9.18.3, ants/v2.6.1, fsl/v6.0.7.7, and the BISON algorithm.<br><br>Bayesian linear regression was used for normative modeling with the PCN toolkit/v0.35. All linear models were processed in R/4.1.2 using the lm function. Spectral clustering was performed with the Spectrum/v1.1 package in R/4.1.2. SuStaIn disease modeling was performed with the pySuStaIn package in python/v3.9.8. The neuromaps python package was used for spatial permutation tests and visualization of cortical results. Lasso logistic regression was performed with the sklearn/1.7.2 python package. ComBat harmonization was performed with the neuroharmonize/2.4.5 python package. Code developed for this manuscript is available at https://github.com/CoBrALab/WMH_patho_UKB (DOI: 10.5281/zenodo.18613580)

For manuscripts utilizing custom algorithms or software that are central to the research but not yet described in published literature, software must be made available to editors and reviewers. We strongly encourage code deposition in a community repository (e.g. GitHub). See the Nature Portfolio guidelines for submitting code & software for further information.

Data

Policy information about availability of data

All manuscripts must include a data availability statement. This statement should provide the following information, where applicable:

- Accession codes, unique identifiers, or web links for publicly available datasets
- A description of any restrictions on data availability
- For clinical datasets or third party data, please ensure that the statement adheres to our policy

Data from UK Biobank is publicly available via material transfer agreements (https://www.ukbiobank.ac.uk/enable-your-research/register). Data from ADNI is publicly available via a data-sharing application (https://adni.loni.usc.edu/data-samples/adni-data/#AccessData). Data from PREVENT-AD is publicly available via a data user agreement (https://openpreventad.loris.ca/).

Research involving human participants, their data, or biological material

Policy information about studies with human participants or human data. See also policy information about sex, gender (identity/presentation), and sexual orientation and race, ethnicity and racism.

Reporting on sex and gender	Sex in UK Biobank is acquired from central registry at recruitment, but in some cases updated by the participant. Hence this field may contain a mixture of the sex the NHS had recorded for the participant and self-reported sex.<br><br>Sex was used as a covariate for the normative modeling and for the linear models comparing disease groups. Sex was used as a variable of interest by assessing the reproducibility of our spatial patterns between males and females, by investigating sex effects in WMH pathophysiology, and by investigating sex by group interactions in disease effects.
Reporting on race, ethnicity, or other socially relevant groupings	We did not use any socially constructed or socially relevant variables in our analyses.
Population characteristics	Details are presented in Supplementary Table 1 for UK Biobank and Supplementary Table 2 for ADNI.<br>The UK Biobank dataset was between 45 and 81 years old (average of 63.52 and SD of 7.49) with 53.4% females. The ADNI dataset was between 51 and 93 years old (average of 73.41 and SD of 8.44) with 58% females.
Recruitment	UK Biobank investigators sent postal invitations to 9,238,453 individuals registered with the UK's National Health Service who were aged 40–69 years and lived within approximately 25 miles (40 km) of one of 22 assessment centers located throughout England, Wales, and Scotland. For ADNI3, the Coordinating Center managed recruitment activities (national newspaper and radio coverage; local TV and newspaper coverage; and search engine, website, social media and newspaper advertisements) to connect potential participants directly with local research sites.
Ethics oversight	The UK Biobank (UKB) dataset was approved by the North West Multicenter Research Ethics Committee (United Kingdom). The Alzheimer's Disease Neuroimaging Initiative (ADNI) dataset was approved by the institutional review board at each participating institution. The Pre-symptomatic Evaluation of Experimental or Novel Treatments for Alzheimer Disease (PREVENT-AD) was approved by the institutional review board at McGill University. All participants gave written informed consent prior to participation in the studies.

Note that full information on the approval of the study protocol must also be provided in the manuscript.

Field-specific reporting

Please select the one below that is the best fit for your research. If you are not sure, read the appropriate sections before making your selection.

☒ Life sciences ☐ Behavioural & social sciences ☐ Ecological, evolutionary & environmental sciences

For a reference copy of the document with all sections, see nature.com/documents/nr-reporting-summary-flat.pdf

Life sciences study design

All studies must disclose on these points even when the disclosure is negative.

Sample size	We did not make use of statistical methods to determine sample size. Samples were chosen based on data availability and effects were replicated in other datasets where possible.
Data exclusions	See Supplementary Figure 1 for step-by-step exclusions. We excluded participants that did not have all MRI acquisitions necessary, that had missing age or sex data, that had motion artifacts in any MRI acquisition, that failed MRI processing steps, and that had a multple sclerosis diagnosis (to exclude white matter lesions from WMHs).
Replication	We performed sensitivity analyses for the spatial clustering step, confirming the robustness of the results in people with different total WMH volumes and across males and females. We replicated our finding of disease effects using UK Biobank diagnosis data, UK Biobank genetic risk data, ADNI diagnosis data, and ADNI biomarker data (Figure 4).

Randomization	This study did not contain different experimental conditions.
Blinding	This study did not contain different experimental conditions so blinding was not possible. The staff involved in data collection were notinvolved in data analysis.

Behavioural & social sciences study design

All studies must disclose on these points even when the disclosure is negative.

Study description	*Briefly describe the study type including whether data are quantitative, qualitative, or mixed-methods (e.g. qualitative cross-sectional, quantitative experimental, mixed-methods case study).*
Research sample	*State the research sample (e.g. Harvard university undergraduates, villagers in rural India) and provide relevant demographic information (e.g. age, sex) and indicate whether the sample is representative. Provide a rationale for the study sample chosen. For studies involving existing datasets, please describe the dataset and source.*
Sampling strategy	*Describe the sampling procedure (e.g. random, snowball, stratified, convenience). Describe the statistical methods that were used to predetermine sample size OR if no sample-size calculation was performed, describe how sample sizes were chosen and provide a rationale for why these sample sizes are sufficient. For qualitative data, please indicate whether data saturation was considered, and what criteria were used to decide that no further sampling was needed.*
Data collection	*Provide details about the data collection procedure, including the instruments or devices used to record the data (e.g. pen and paper, computer, eye tracker, video or audio equipment) whether anyone was present besides the participant(s) and the researcher, and whether the researcher was blind to experimental condition and/or the study hypothesis during data collection.*
Timing	*Indicate the start and stop dates of data collection. If there is a gap between collection periods, state the dates for each sample cohort.*
Data exclusions	*If no data were excluded from the analyses, state so OR if data were excluded, provide the exact number of exclusions and the rationale behind them, indicating whether exclusion criteria were pre-established.*
Non-participation	*State how many participants dropped out/declined participation and the reason(s) given OR provide response rate OR state that no participants dropped out/declined participation.*
Randomization	*If participants were not allocated into experimental groups, state so OR describe how participants were allocated to groups, and if allocation was not random, describe how covariates were controlled.*

Ecological, evolutionary & environmental sciences study design

All studies must disclose on these points even when the disclosure is negative.

Study description	*Briefly describe the study. For quantitative data include treatment factors and interactions, design structure (e.g. factorial, nested, hierarchical), nature and number of experimental units and replicates.*
Research sample	*Describe the research sample (e.g. a group of tagged Passer domesticus, all Stenocereus thurberi within Organ Pipe Cactus National Monument), and provide a rationale for the sample choice. When relevant, describe the organism taxa, source, sex, age range and any manipulations. State what population the sample is meant to represent when applicable. For studies involving existing datasets, describe the data and its source.*
Sampling strategy	*Note the sampling procedure. Describe the statistical methods that were used to predetermine sample size OR if no sample-size calculation was performed, describe how sample sizes were chosen and provide a rationale for why these sample sizes are sufficient.*
Data collection	*Describe the data collection procedure, including who recorded the data and how.*
Timing and spatial scale	*Indicate the start and stop dates of data collection, noting the frequency and periodicity of sampling and providing a rationale for these choices. If there is a gap between collection periods, state the dates for each sample cohort. Specify the spatial scale from which the data are taken*
Data exclusions	*If no data were excluded from the analyses, state so OR if data were excluded, describe the exclusions and the rationale behind them, indicating whether exclusion criteria were pre-established.*
Reproducibility	*Describe the measures taken to verify the reproducibility of experimental findings. For each experiment, note whether any attempts to repeat the experiment failed OR state that all attempts to repeat the experiment were successful.*
Randomization	*Describe how samples/organisms/participants were allocated into groups. If allocation was not random, describe how covariates were controlled. If this is not relevant to your study, explain why.*
Blinding	*Describe the extent of blinding used during data acquisition and analysis. If blinding was not possible, describe why OR explain why blinding was not relevant to your study.*

Did the study involve field work? ☐ Yes ☐ No

Field work, collection and transport

Field conditions	*Describe the study conditions for field work, providing relevant parameters (e.g. temperature, rainfall).*
Location	*State the location of the sampling or experiment, providing relevant parameters (e.g. latitude and longitude, elevation, water depth).*
Access & import/export	*Describe the efforts you have made to access habitats and to collect and import/export your samples in a responsible manner and in compliance with local, national and international laws, noting any permits that were obtained (give the name of the issuing authority, the date of issue, and any identifying information).*
Disturbance	*Describe any disturbance caused by the study and how it was minimized.*

Reporting for specific materials, systems and methods

We require information from authors about some types of materials, experimental systems and methods used in many studies. Here, indicate whether each material, system or method listed is relevant to your study. If you are not sure if a list item applies to your research, read the appropriate section before selecting a response.

Materials & experimental systems

n/a	Involved in the study
☒	☐ Antibodies
☒	☐ Eukaryotic cell lines
☒	☐ Palaeontology and archaeology
☒	☐ Animals and other organisms
☒	☐ Clinical data
☒	☐ Dual use research of concern
☒	☐ Plants

Methods

n/a	Involved in the study
☒	☐ ChIP-seq
☒	☐ Flow cytometry
☐	☒ MRI-based neuroimaging

Antibodies

Antibodies used	*Describe all antibodies used in the study; as applicable, provide supplier name, catalog number, clone name, and lot number.*
Validation	*Describe the validation of each primary antibody for the species and application, noting any validation statements on the manufacturer's website, relevant citations, antibody profiles in online databases, or data provided in the manuscript.*

Eukaryotic cell lines

Policy information about cell lines and Sex and Gender in Research

Cell line source(s)	*State the source of each cell line used and the sex of all primary cell lines and cells derived from human participants or vertebrate models.*
Authentication	*Describe the authentication procedures for each cell line used OR declare that none of the cell lines used were authenticated.*
Mycoplasma contamination	*Confirm that all cell lines tested negative for mycoplasma contamination OR describe the results of the testing for mycoplasma contamination OR declare that the cell lines were not tested for mycoplasma contamination.*
Commonly misidentified lines (See ICLAC register)	*Name any commonly misidentified cell lines used in the study and provide a rationale for their use.*

Palaeontology and Archaeology

Specimen provenance	*Provide provenance information for specimens and describe permits that were obtained for the work (including the name of the issuing authority, the date of issue, and any identifying information). Permits should encompass collection and, where applicable, export.*
Specimen deposition	*Indicate where the specimens have been deposited to permit free access by other researchers.*
Dating methods	*If new dates are provided, describe how they were obtained (e.g. collection, storage, sample pretreatment and measurement), where they were obtained (i.e. lab name), the calibration program and the protocol for quality assurance OR state that no new dates are*

provided.

☐ Tick this box to confirm that the raw and calibrated dates are available in the paper or in Supplementary Information.

Ethics oversight	*Identify the organization(s) that approved or provided guidance on the study protocol, OR state that no ethical approval or guidance was required and explain why not.*

Note that full information on the approval of the study protocol must also be provided in the manuscript.

Animals and other research organisms

Policy information about studies involving animals; ARRIVE guidelines recommended for reporting animal research, and Sex and Gender in Research

Laboratory animals	*For laboratory animals, report species, strain and age OR state that the study did not involve laboratory animals.*
Wild animals	*Provide details on animals observed in or captured in the field; report species and age where possible. Describe how animals were caught and transported and what happened to captive animals after the study (if killed, explain why and describe method; if released, say where and when) OR state that the study did not involve wild animals.*
Reporting on sex	*Indicate if findings apply to only one sex; describe whether sex was considered in study design, methods used for assigning sex. Provide data disaggregated for sex where this information has been collected in the source data as appropriate; provide overall numbers in this Reporting Summary. Please state if this information has not been collected. Report sex-based analyses where performed, justify reasons for lack of sex-based analysis.*
Field-collected samples	*For laboratory work with field-collected samples, describe all relevant parameters such as housing, maintenance, temperature, photoperiod and end-of-experiment protocol OR state that the study did not involve samples collected from the field.*
Ethics oversight	*Identify the organization(s) that approved or provided guidance on the study protocol, OR state that no ethical approval or guidance was required and explain why not.*

Note that full information on the approval of the study protocol must also be provided in the manuscript.

Clinical data

Policy information about clinical studies

All manuscripts should comply with the ICMJE guidelines for publication of clinical research and a completed CONSORT checklist must be included with all submissions.

Clinical trial registration	*Provide the trial registration number from ClinicalTrials.gov or an equivalent agency.*
Study protocol	*Note where the full trial protocol can be accessed OR if not available, explain why.*
Data collection	*Describe the settings and locales of data collection, noting the time periods of recruitment and data collection.*
Outcomes	*Describe how you pre-defined primary and secondary outcome measures and how you assessed these measures.*

Dual use research of concern

Policy information about dual use research of concern

Hazards

Could the accidental, deliberate or reckless misuse of agents or technologies generated in the work, or the application of information presented in the manuscript, pose a threat to:

No	Yes	
☐	☐	Public health
☐	☐	National security
☐	☐	Crops and/or livestock
☐	☐	Ecosystems
☐	☐	Any other significant area

Experiments of concern

Does the work involve any of these experiments of concern:

No	Yes	
☐	☐	Demonstrate how to render a vaccine ineffective
☐	☐	Confer resistance to therapeutically useful antibiotics or antiviral agents
☐	☐	Enhance the virulence of a pathogen or render a nonpathogen virulent
☐	☐	Increase transmissibility of a pathogen
☐	☐	Alter the host range of a pathogen
☐	☐	Enable evasion of diagnostic/detection modalities
☐	☐	Enable the weaponization of a biological agent or toxin
☐	☐	Any other potentially harmful combination of experiments and agents

Plants

Seed stocks	*Report on the source of all seed stocks or other plant material used. If applicable, state the seed stock centre and catalogue number. If plant specimens were collected from the field, describe the collection location, date and sampling procedures.*
Novel plant genotypes	*Describe the methods by which all novel plant genotypes were produced. This includes those generated by transgenic approaches, gene editing, chemical/radiation-based mutagenesis and hybridization. For transgenic lines, describe the transformation method, the number of independent lines analyzed and the generation upon which experiments were performed. For gene-edited lines, describe the editor used, the endogenous sequence targeted for editing, the targeting guide RNA sequence (if applicable) and how the editor was applied.*
Authentication	*Describe any authentication procedures for each seed stock used or novel genotype generated. Describe any experiments used to assess the effect of a mutation and, where applicable, how potential secondary effects (e.g. second site T-DNA insertions, mosiacism, off-target gene editing) were examined.*

ChIP-seq

Data deposition

☐ Confirm that both raw and final processed data have been deposited in a public database such as GEO.

☐ Confirm that you have deposited or provided access to graph files (e.g. BED files) for the called peaks.

Data access links<br>*May remain private before publication.*	*For "Initial submission" or "Revised version" documents, provide reviewer access links. For your "Final submission" document, provide a link to the deposited data.*
Files in database submission	*Provide a list of all files available in the database submission.*
Genome browser session<br>(e.g. UCSC)	*Provide a link to an anonymized genome browser session for "Initial submission" and "Revised version" documents only, to enable peer review. Write "no longer applicable" for "Final submission" documents.*

Methodology

Replicates	*Describe the experimental replicates, specifying number, type and replicate agreement.*
Sequencing depth	*Describe the sequencing depth for each experiment, providing the total number of reads, uniquely mapped reads, length of reads and whether they were paired- or single-end.*
Antibodies	*Describe the antibodies used for the ChIP-seq experiments; as applicable, provide supplier name, catalog number, clone name, and lot number.*
Peak calling parameters	*Specify the command line program and parameters used for read mapping and peak calling, including the ChIP, control and index files used.*
Data quality	*Describe the methods used to ensure data quality in full detail, including how many peaks are at FDR 5% and above 5-fold enrichment.*
Software	*Describe the software used to collect and analyze the ChIP-seq data. For custom code that has been deposited into a community repository, provide accession details.*

Flow Cytometry

Plots

Confirm that:

- [ ] The axis labels state the marker and fluorochrome used (e.g. CD4-FITC).
- [ ] The axis scales are clearly visible. Include numbers along axes only for bottom left plot of group (a 'group' is an analysis of identical markers).
- [ ] All plots are contour plots with outliers or pseudocolor plots.
- [ ] A numerical value for number of cells or percentage (with statistics) is provided.

Methodology

Sample preparation	*Describe the sample preparation, detailing the biological source of the cells and any tissue processing steps used.*
Instrument	*Identify the instrument used for data collection, specifying make and model number.*
Software	*Describe the software used to collect and analyze the flow cytometry data. For custom code that has been deposited into a community repository, provide accession details.*
Cell population abundance	*Describe the abundance of the relevant cell populations within post-sort fractions, providing details on the purity of the samples and how it was determined.*
Gating strategy	*Describe the gating strategy used for all relevant experiments, specifying the preliminary FSC/SSC gates of the starting cell population, indicating where boundaries between "positive" and "negative" staining cell populations are defined.*

- [ ] Tick this box to confirm that a figure exemplifying the gating strategy is provided in the Supplementary Information.

Magnetic resonance imaging

Experimental design

Design type	Correlational study design
Design specifications	Not applicable
Behavioral performance measures	No measures of behavior were examined in this study

Acquisition

Imaging type(s)	Structural (T1 and T2-FLAIR), diffusion-weighted MRI, susceptibility-weighted MRI
Field strength	3T
Sequence & imaging parameters	T1-weighted: Sagittal 3D MPRAGE; in-plane acceleration factor (R) = 2; inversion time (TI) = 880 ms; repetition time (TR) = 2000 ms; resolution = 1 x 1 x 1 mm<br>T2-weighted Fluid-attenuated inversion recovery (FLAIR): Sagittal 3D SPACE; R = 2; partial Fourier (PF) = 7/8; fat saturation; TI = 1800 ms; TR = 5000 ms; elliptical k-space scanning; resolution = 1.05 x 1 x 1 mm<br>Diffusion-weighted imaging (DWI): SE-EPI; multiband factor (MB) = 3; R = 1; TE = 92 ms; TR = 3600 ms; PF = 6/8; fat saturation; b-values: 5 x b = 0 s/mm2, 50 x b = 1000 s/mm2, 50 x b = 2000 s/mm2 (100 distinct diffusion directions); phase-encoding reversed data acquired; resolution = 2 x 2 x 2 mm<br>Susceptibility-weighted imaging (SWI): Axial 3D GRE; R = 2, PF = 7/8; TE1 = 9.4 ms; TE2 = 20 ms; TR = 27 ms; resolution = 0.8 x 0.8 x 3 mm
Area of acquisition	Whole brain

Diffusion MRI [x] Used [ ] Not used

Preprocessing

Preprocessing software	MRI data was processed using minc-toolkit/v1.9.18.3, ants/v2.6.1, fsl/v6.0.7.7, and the BISON algorithm.
Normalization	All modalities were aligned at the subject level by performing rigid registration of other modalities (FLAIR, diffusion, and susceptibility) to T1w subject space using the Advanced Normalization Tools (ANTs) software (antsRegistration_affine_SyN.sh script from minc-toolkit-extras https://github.com/CoBrALab/minc-toolkit-extras). We then performed multispectral non-linear registration to the study-specific templates using preprocessed T1w images and fractional anisotropy maps supersampled to 1 mm (using the NonLocalSuperResolution command from ANTs) isotropic as inputs. The ADNI template was non-linearly registered to the UKB template. All microstructural maps were transformed in UKB space using 4th-order B-spline interpolation at a 2 mm isotropic resolution to match the diffusion-weighted imaging resolution (of note, the

susceptibility-weighted imaging acquisition had an original resolution of 0.8 x 0.8 x 3 mm). The rich spatial contrast of fractional anisotropy maps allowed for increased registration accuracy of white matter tracts, a crucial aspect for our application.

Normalization template

Study-specific UK Biobank template

Noise and artifact removal

T1w and FLAIR images were denoised (minc_anlm), corrected for N3 inhomogeneities (nu_correct), and intensity normalized (volume_pol). Brain masks were calculated using the BEaST algorithm. The multi-shell diffusion-weighted images were corrected for susceptibility artifacts using the topup FSL command, eddy currents, head motion, and outliers using the eddy command, and gradient distortions. The susceptibility-weighted images were saved as magnitude and phase images separately for each coil. Magnitude data was combined across coils using a sum-of-squares calculation, and from this data, T2* is calculated as the inverse of the log ratio of the two echo times scaled by the echo time difference. Phase data was combined across coils using MCPC-3D-S, which removes phase cancellation artifacts from each echo. This data underwent phase unwrapping using a Laplacian algorithm, background field removal using V-SHARP, and brain mask erosion to exclude voxels with low phase reliability. Dipole inversion using iLSQR was then used to estimate quantitative susceptibility mapping (QSM) maps, which were further normalized by the subject-wise median value in the ventricles.

Volume censoring

Not applicable

Statistical modeling & inference

Model type and settings

All linear models were processed in R/4.1.2 using the lm function, scaling all continuous variables to obtain standardized beta coefficients. p-values were corrected using the false discovery rate (FDR)44 on a per-analysis basis unless otherwise indicated.
For spatial clustering, averaged WMH pathophysiology maps were used as inputs to a spectral clustering algorithm using the Spectrum package in R. We calculated the variance explained (R2) in the maps of between-subject averaged WMH pathophysiology by the spatial clustering solution or each microstructural metric using ANOVAs. We calculated the Dice similarity coefficient (DSC) overlaps between our derived spatial cluster and other often-used parcellations. We then calculated region-of-interest measures of WMH pathophysiology by computing the median pathophysiological value for each metric within WMHs in each spatial cluster.
To model the temporal progression of WMH pathophysiological events, we used the Subtype and Stage Inference (SuStaIn) technique, specifically the piecewise linear z-score model. SuStaIn was performed using 10-fold cross-validation with 15 startpoints and 10,000 Monte Carlo Markov Chain resamples at each fold. We also modeled the relationships between WMH pathophysiology and volume. Non-linear relationships with log-transformed WMH volume were modeled with 4th-order B-splines. Spearman correlations were calculated for associations between SuStaIn stages and regional WMH volumes.
Sex differences in WMH measures were investigated using linear models 1) for regional WMH volumes, correcting for age, and 2) for WMH pathophysiological estimates, additionally correcting for region-specific WMH volume.
For associations with diseases, we compared WMHs between cases and controls using linear models while covarying for age and sex. Each analysis was repeated with a group by sex interaction term to investigate sex differences in the reported effects. Each effect size pattern was correlated to all other patterns with Pearson's correlations, excluding modalities that were not present for the correlations with ADNI patterns. Hierarchical clustering was performed on the resulting correlation matrix using the hclust function in R.
For classifying stroke from dementia with WMH predictors, we used the LogisticRegression function from sklearn in python (parameters: L1 penalty, C = 1, saga solver, balanced class weights (to address class imbalances), and max_iter = 1000).
For associations with grey matter pathology, we computed Pearson correlations between the cortical connectivity maps and averaged PET maps, performed "spin tests" to calculate 10,000 permutation-based nulls preserving the spatial autocorrelation, and derived p-values using the neuromaps package in Python. p-values were corrected with Bonferroni instead of FDR due to the lower amount of comparisons.

Effect(s) tested

See above

Specify type of analysis: ☐ Whole brain ☐ ROI-based ☒ Both

Statistic type for inference
(See Eklund et al. 2016)

Group comparisons are performed at the ROI level.

Correction

FDR or Bonfferoni correction was applied where applicable

Models & analysis

n/a	Involved in the study
☒	☐ Functional and/or effective connectivity
☒	☐ Graph analysis
☒	☐ Multivariate modeling or predictive analysis

Functional and/or effective connectivity

Report the measures of dependence used and the model details (e.g. Pearson correlation, partial correlation, mutual information).

Graph analysis

Report the dependent variable and connectivity measure, specifying weighted graph or binarized graph, subject- or group-level, and the global and/or node summaries used (e.g. clustering coefficient, efficiency, etc.).

Multivariate modeling and predictive analysis

Specify independent variables, features extraction and dimension reduction, model, training and evaluation metrics.
